# Automatic Cleaning of Time Series Data in Rural Internet of Things Ecosystems That Use Nomadic Gateways

**DOI:** 10.3390/s25010189

**Published:** 2025-01-01

**Authors:** Jerzy Dembski, Agata Kołakowska, Bogdan Wiszniewski

**Affiliations:** Faculty of Electronics, Telecommunications and Informatics, Gdańsk University of Technology, Narutowicza 11/12, 80-233 Gdańsk, Poland; jerzy.dembski@pg.edu.pl (J.D.); agata.kolakowska@pg.edu.pl (A.K.)

**Keywords:** intelligent sensors, energy and bandwidth constraints, nomadic computing

## Abstract

A serious limitation to the deployment of IoT solutions in rural areas may be the lack of available telecommunications infrastructure enabling the continuous collection of measurement data. A nomadic computing system, using a UAV carrying an on-board gateway, can handle this; it leads, however, to a number of technical challenges. One is the intermittent collection of data from ground sensors governed by weather conditions for the UAV measurement missions. Therefore, each sensor should be equipped with software that allows for the cleaning of collected data before transmission to the fly-over nomadic gateway from erroneous, misleading, or otherwise redundant data—to minimize their volume and fit them in the limited transmission window. This task, however, may be a barrier for end devices constrained in several ways, such as limited energy reserve, insufficient computational capability of their MCUs, and short transmission range of their RAT modules. In this paper, a comprehensive approach to these problems is proposed, which enables the implementation of an anomaly detector in time series data with low computational demand. The proposed solution uses the analysis of the physics of the measured signals and is based on a simple anomaly model whose parameters can be optimized using popular AI techniques. It was validated during a full 10-month vegetation period in a real Rural IoT system deployed by Gdańsk Tech.

## 1. Introduction

The impressive development of artificial intelligence methods, supported by cloud computing, creates numerous opportunities for implementing intelligent solutions in various areas involving remote sensing, in  particular those based on satellite Earth Observation (EO). Unfortunately, when it comes to measuring soil parameters, they need in situ measurements for reference and calibration, since inconsistency and lack of precision of EO data can lead to inaccuracies in crop monitoring and precision agriculture [1]. An alternative to EO in such applications may be IoT, with numerous sensors distributed over often large areas and forming a measurement ecosystem. However, in such a case, there is a problem with the availability of telecommunications infrastructure that would ensure the transmission of data collected by individual sensors to some computing cloud instance, where they could be processed in order to perform various tasks [2]—from simple visualization of the spatial distribution of measured soil parameters, through data classification in order to identify and localize specific phenomena in the monitored area, to tasks of predicting the course of vegetation processes. But in remote locations over vast areas, telecommunications infrastructure may not be available. Furthermore, end devices with constraints on power reserves and available bandwidth will not be able to interact with a cloud computing infrastructure designed to perform the aforementioned tasks. In this paper, we refer to such sensor ecosystems as rural IoTs.

**Figure 1:**
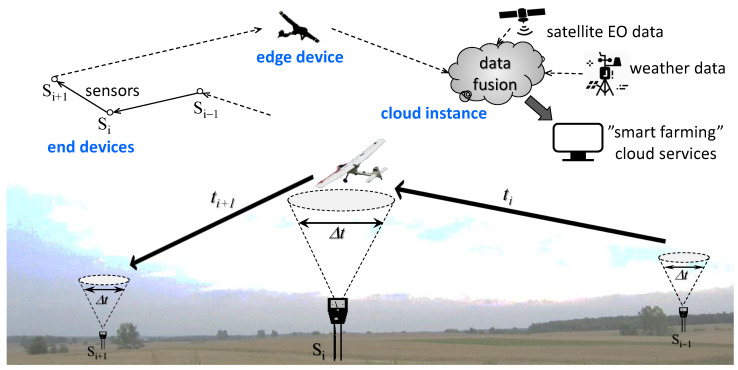
Nomadic computing in rural areas. Measurement sensors scattered over a large area without access to telecommunications infrastructure need an intermediary in the form of a mobile gateway carried by a UAV. Due to the limited Δt fly-over window, the transmitted data samples should not contain redundant, erratic, or otherwise misleading data.

The underlying concept is to collect measurement data from sensor devices by an Unmanned Aerial Vehicle (UAV) piloted from the ground or operating autonomously—a sort of a “go-between”, capable of delivering chunks of data from end devices to the cloud. Individual end (sensor) devices are equipped with a Radio Access Technology (RAT) unit communicating with a nomadic gateway on-board the UAV flying over them. As outlined in Figure 1, the latter constitutes an edge component of the end–edge–cloud setting [3].

The ultimate objective of the research presented in this paper is the effective use of the available transmission window Δt by reducing the volume of data transmitted aboard the UAV and aggregate the data to the minimum possible. The presence of erratic or otherwise anomalous samples may distort the original correct signal waveform after extracting it later on from the aggregated form by the computing cloud. Based on the tests of a pilot version of a rural IoT system implemented by us in a radius of up to 35 km from the computing cloud at Gdańsk Tech, we were able to develop and validate an effective methodology for dealing with the scarcity of measurement data implied by a very low rate of collecting soil data, spread over the entire 10-month vegetation period, and their intermitted flow due to weather constraints for UAV missions. The key to this achievement was the adoption of a comprehensive, physics-informed approach to developing an anomaly detector instead of the descriptive approach, commonly used in signal analysis; we present it further in this paper in detail.

Surprisingly, we did not find in the literature any attempt to introduce such physics-informed analysis of time series data in IoT that would directly leverage the sensor’s capability—only solutions for collecting data for further processing, e.g., in HUMS systems [4]. Although physics (if considered at all) is used for collecting experimental (measurement) data that embody the underlying phenomena, the objective is to directly introduce observational biases to already pre-trained models—rather then explain and parametrize motifs related to such phenomena, which may appear in the signal’s waveform [5]. A dominant approach seems to be all the same: collect whatever data can be collected (often a stream assuming their constant flow, e.g., video surveillance systems or social media), and then process them on some central unit of a preferably unlimited computing power, e.g., a cloud instance. This favors descriptive approaches—large amounts of data are processed with Deep Learning (DL) models to analyze the signal waveform in order to discover its underlying physics if possible, or even when analyzing socio-economic processes.

The main beneficiaries of the solution proposed in this paper are manufacturers of various types of IoT sensors which, due to their limitations, have limited capabilities of analyzing and improving the quality of the recorded data. Such sensors may be designed as cheap but smart end devices that can work without any external support, often functioning as disposable and durable units. In addition, the use of such sensors in IoT ecosystems, where UAVs carrying an edge gateway can collect data from areas without operational telecommunications infrastructure, creates opportunities for implementing various useful scenarios—beyond agriculture and forestry, including river monitoring for flood alerts and warnings, or monitoring large-scale disaster areas that have been temporarily deprived of telecommunications.

The structure of this paper is as follows: In Section 2, the declarative approach to time series analysis is contrasted with the comprehensive one, and the advantage of the latter is argued based on the physical properties of soil signals. These properties enable the identification and explanation of several classes of anomalies, which are formally defined in Section 3 using a parametrized model and detection criteria implemented by the anomaly detector. A method for optimizing model parameters is demonstrated in Section 4. Experimental results reported in Section 5 indicate that heuristic values of the anomaly model parameters set by human interpreters can be further improved using popular AI techniques. Section 6 summarizes the contribution of this paper and the importance of the key findings reported.

## 2. Identification of Anomalies in Soil Data

Each sensor was programmed to periodically measure four parameters, namely, soil temperature T, soil moisture M (a volumetric soil water content), soil acidity/alkalinity pH, and solar irradiance PV (on the PV cell), as listed in Table 1. All collected data were stored as time series of samples.

For collecting measurement data from sensors, we used both a stationary (ground) and a nomadic (on-board UAV) LoRaWAN gateway, acting as alternative edge devices for testing purposes. Each gateway listened in the relevant radio band, received frames, and forwarded them to network server NS, which were subsequently forwarded to the application server AS for decryption and loading to the data storage DS. For the stationary gateway NS, AS and DS were implemented in the cloud, whereas for the nomadic gateway, NS and AS were running on an on-board Raspberry Pi computer, and DS was a removable memory card. After landing the UAV, the content of the card was uploaded manually to the cloud. Data collected from all sensors were finally stored in the InfluxDB [6] database. From there, all their further processing was performed: data fusion (time series from multiple sensors), data augmentation with data from other sources (meteorological data and satellite images), and various classification and prediction tasks involving computationally demanding ML techniques. These issues, however, are beyond the scope of this paper.

During one growing season, about 12 MB of raw (uncleaned) data were collected, which included over 175,000 samples of T, M, pH, and PV signals [7]. We tried to interpret them in different ways to identify anomalies that (1) were meaningful in terms of disturbing the true waveform of each measured soil signal and (2) could be effectively eliminated by sensors lacking the computational resources required for advanced signal analysis.

### 2.1. Descriptive Approach

At first glance, it might seem that a descriptive representation of the measured signal waveform should reveal all visible disturbances in its correct course. We can search for these representations in two ways, as advocated in the literature: by statistical analysis of the sequence of samples [8] or by searching for visual motifs and change points in their waveforms [9]. In our case, each of them gave ambiguous results, making them unworthy of implementation in the sensor code. Moreover, some of them were too computationally expensive for a given constrained end device [10,11]. But even if implemented, they could introduce biases into the analysis, because, as we will illustrate with examples further in this paper, unusual motifs or change points can often represent the correct course of the monitored phenomenon [12].

Statistical analysis techniques decompose the signal into seasonal, trend, and residue parts. With soil data, several challenges arise, due to the non-stationarity of some signal mean and variance, as well as seasonality and trends fluctuating irregularly over intervals with a length difficult to grasp. Practically, only the soil temperature signal T showed a clear trend during the day, repeating regularly between each two consecutive sunrises. Contrary to T, the statistical decomposition of the soil moisture signal M did not reveal any significant recurring patterns along the entire growing season—either in trend or in seasonality. The acidity signal pH showed even less of the above, remaining at a constant level for most of the season.

In turn, visual motif and change point search attempts to classify some general “common sense” patterns, and then identify them in the analyzed signal waveform as deviations from the course considered normal. For example, in [9], one such common sense classification was proposed, distinguishing *point*, *contextual*, and *collective/pattern* anomalies. Such a classification for our application turned out to be useless, as it assumes that during signal analysis, the sensor knows the normal (correct) signal course. Certainly, such an approach could be more suitable for the task of detecting anomalies in the course of a monitored base process, rather than the monitoring process itself, which is secondary to the former one. In other words, our task is not to search for anomalies in soil processes, but to eliminate erratic data that are the result of anomalies occurring in the measurement process. One example of this problem is power outages, discussed by us further in this paper; they may sporadically occur in sensors and interfere with the time-stamping of samples received by a nomadic gateway.

Deep Learning (DL) and Big Data methods, in turn, can be placed at the opposite end of the spectrum. Their contemporary success is based on the ability to discover and analyze patterns observed in the data generated by processes whose physics is not known or cannot be formally described, such as in the case of socio-demographic or medical data [13]. A condition for success, however, is to guarantee access to a sufficiently large amount of raw (measurement) data [14]. Unfortunately, these approaches would come down to completely unrealistic costs of implementing measurement campaigns of soil parameters, when huge volumes of measurement data from numerous sensors and many growing seasons may be required to train DL models [15].

### 2.2. Comprehensive Approach

An attempt to construct an effective mechanism for the automatic detection and classification of anomalies in our soil time series data encountered the well-known “chicken and egg” dilemma—whether we should first collect an appropriate amount of data and then discover some characteristic patterns in it, or determine what patterns are meaningful and then look for them in the analyzed set. However, the problem with deploying a rural IoT ecosystem, as shown in Figure 1, is the relatively high cost of collecting data to begin with; i.e., before placing sensors in the field, they have to be programmed to process the data collected locally to the form that would fit in the available Δt time window. Thus, anomalies had to be identified and understood first.

Let us start by considering the meaning of the basic physical processes that determine the course of the signals recorded by our sensors:

- Soil temperature (T): Solar heat is gradually accumulated in the soil from sunrise and radiated out after sunset. In consequence, the signal slowly increases until sunset and decreases afterwards, thus exhibiting a strong daily trend and seasonality.
- Soil moisture (M): The physical properties of soil moisture measured in a depth of about 0.5 m indicate significantly extended seasonality intervals—their analysis may require even decades [16]. In consequence, both the trend and seasonality of the signal are hard to capture. Moreover, heavy rainfalls combined with soil/terrain conditions and the location of the sensor may result in temporary flooding of the measurement probe. Hence, over a period of several days, when a single sensor makes its daily measurements, one can only expect slow changes in the signal with varying random trends.
- Soil acidity/alkalinity (pH): The signal does not change much from month to month or even year to year [17]. This is due to the fact that soil solids dissolve very slowly in the soil solution and gradually supplement the microelements that are crucial for vegetation. Consequently, pH should not be considered seasonal, as it may show at most some barely noticeable changes possibly correlated with changes in soil moisture M.
- Solar irradiance (PV): The cell produces a nominal maximum voltage, which rises/drops logarithmically from/to a near-zero value for solar irradiance above/below a certain minimal threshold, while remaining high and almost unchanged at higher irradiance levels [18]. In consequence, the signal should exhibit no trend and strong daily seasonality (sunrise/sunset cycles), but possibly with a significant residual component depending on the actual charge level of the power supply battery.

Below, we interpret patterns that could be observed in the waveforms of our soil signals, which, although named descriptively, can be explained in accordance with their underlying physics. This interpretation is crucial for determining the parameters of our anomaly model used for data cleaning in Section 3, and generating the synthetic data used for optimizing its parameters in Section 4, in a way that does not introduce biases or misinterpretations that may result from adopting only the descriptive approaches described above.

#### 2.2.1. Missing or Misplaced Samples

**Figure 2:**
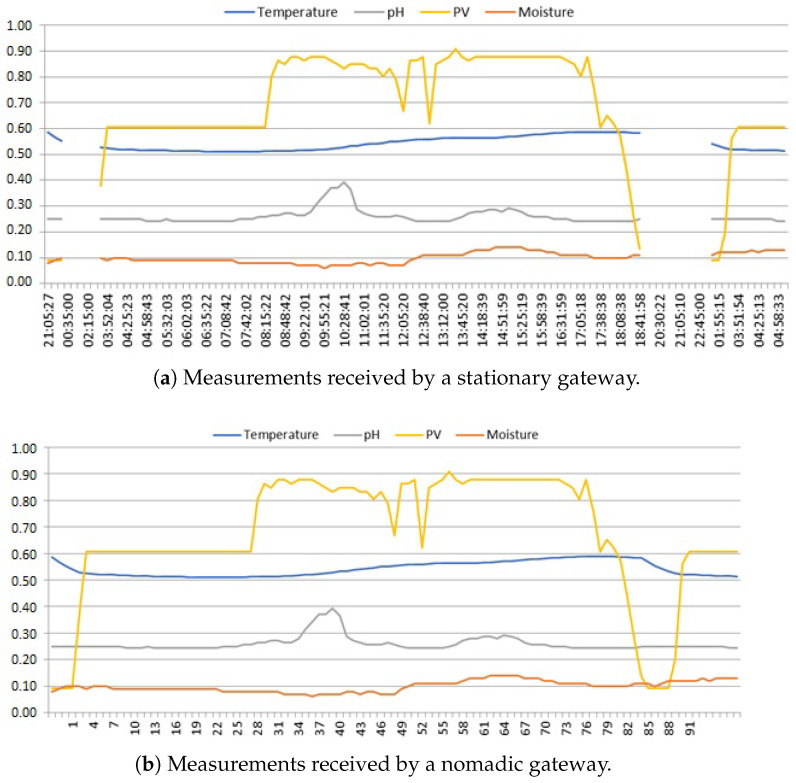
Time series data with power gaps. A nomadic gateway that connects to a sensor irregularly is not able to automatically detect power outages if the latter is not equipped with a continuously powered system clock.

By comparing the same portions of time series data, one received by a stationary gateway and another by its nomadic counterpart, it may be seen in Figure 2 that some samples in between two consecutive sunrises (at about 03:45 h) are missing. The lack of measurements after 19:00 h that day was due to a power outage in the sensor—caused by insufficient charging of its battery to maintain operation after sunset. The time of this event may be known only to the stationary gateway, which has an internal clock with power backup independent of the sensor. Our Arduino-based end devices did not experience that, as they only operated a time counter that stopped when the power went down. Upon power restoration, the timer resumed counting samples from the last stored value. The stationary gateway can record this fact by measuring the silence period of the sensor, whereas its nomadic counterpart receives samples numbered continuously with the counter values. As a result, the  number of daily samples affected by power outages which are received by a nomadic gateway is smaller, and the waveforms of all four signals over time are distorted; note that in Figure 2b, there are only 88 samples recorded in the analyzed 24 h period, instead of the expected ≈133 samples with a 10 min sampling period, as may be seen in Figure 2a. We call this class of anomalies *power gaps*.

Another observed anomaly, in addition to power gaps, is the timer drift, which causes the number of daily samples to vary. The original sampling period was set programmatically to about τ=10 min, but it turned out that it varied from 6 to even 15 min; this phenomenon was most likely related to the location of the end device and its working (operational environment) temperature.

#### 2.2.2. Erratic Samples

According to Table 1, each measured physical value should stay within certain limits known before programming the sensor, as it must be consistent with its underlying physics. Any “out-of-range” sample implies a measurement *absolute error*. For example, the minimal soil temperature T at 0.5 m below the ground level should never drop below 0 °C during the growing season, even if there were occasional ground frosts [19]. On the other hand, a maximum (never-exceeded) value of T could be a daily temperature record for a given area (e.g., the highest daily average temperature of 40 °C ever recorded in Poland since 1928). Next, although the nominal range of soil pH is [0, 14], its typical range of values should vary from 3.0 (strongly acidic forest soils) to 9.0 (highly alkaline arable soils). These figures depend strongly on the local climate (precipitation vs. evaporation characteristics) and historical geological conditions, and are made public by the respective agriculture authority [20]. In turn, the maximum open-circuit voltage of the PV cell is a parameter specified by its producer. Finally, the natural soil moisture depends on the soil type and may range from 10% (sandy soils) to 45% (clay soils), whereas regarding the type of crops and irrigation, it may range from 20% (flowers, shrubs, and trees) to 80% (vegetables) [19]. In rural IoT systems, we assumed the widest possible range, i.e., from 10% to 80%.

**Figure 3:**
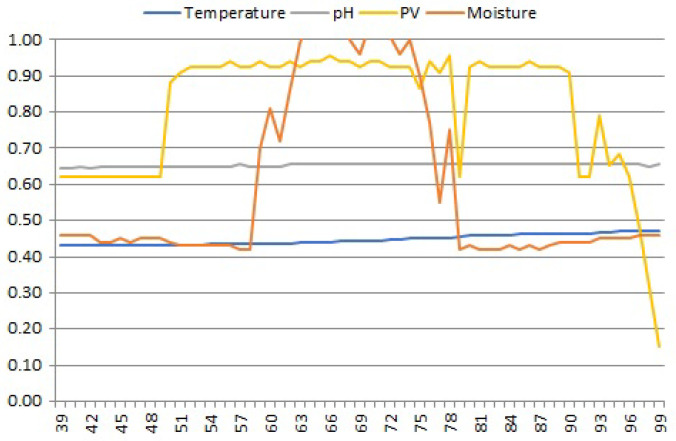
Absolute error in the moisture signal. Anomalous “out-of-range” values most often have internal causes related to the incorrect calibration of the sensor probes of measuring devices.

One example of an absolute error in time series data may be found in Figure 3.

#### 2.2.3. Change Points

**Figure 4:**
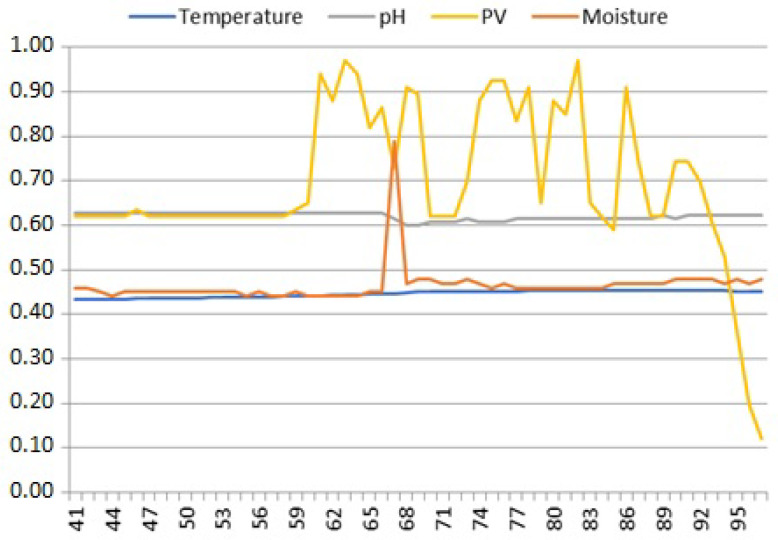
Single peak in the moisture signal. Although some instability of the PV signal is visible, with abrupt changes in the values of its samples 61–91, no other peaks of the moisture signal are present. Apparently, the cause of the single peak observed has its source in the external environment of the moisture sensor probe.

Change points related to abrupt but temporary changes in time series data, often combined with absolute errors, should certainly not occur in slowly changing time series data, as listed in Table 1. One example of such an anomaly is shown in Figure 4, where the value of sample 67 of the moisture signal temporarily increased to the maximum correct value, while the values of the neighboring samples, 66 and 68, did not deviate from the signal trend in the entire measurement interval (08:00–18:22 h). Such changes occurring in time series of soil data are referred to by us as *peaks*.The reason for “peaks” in slowly changing soil parameter values is uncertain; a possible cause of such temporary out-of-trend changes may be some external electrical interference in the operation of analog sensor probes.

**Figure 5:**
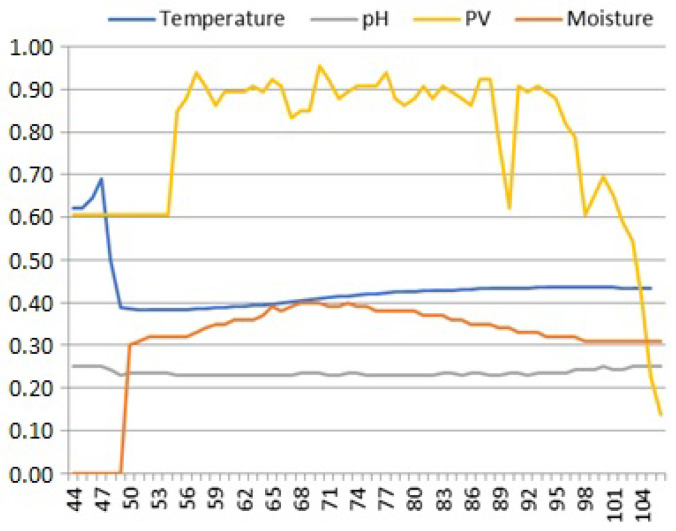
Jumps in the temperature and moisture signals. Their occurrence in slowly changing signals (see Table 1) mean that, for the rest of the daily period, either a given soil sensor probe was turned on or reset or stopped working for some internal reason.

Plots of the solar irradiance (PV) also exhibit characteristic change points indicating daily changes in permanence of its time series data. According to the open-circuit voltage characteristics of a PV cell, its signal changes are abrupt: the voltage jumps up after sunrise, remains high until sunset, and then drops down and stays low until the next sunrise. Such abrupt and durable change points have been classified by us as *jumps*; given the physical properties of the solar irradiance PV signal specified in Table 1, voltage jumps should not be considered anomalies, as data on both sides of them are normal voltage levels. However, jumps in M and T signals in Figure 5 are certainly anomalies—these signals should not change abruptly. Moreover, comparison of the respective values on both sides of the “jumps” in M and T indicates that either the left or right side contains incorrect data. For M, it is the zero value of samples 44–49 preceding the jump to values within the correct range starting from sample 50. On the other hand, although samples 44–49 of T have values within the allowed range, they differ significantly from the trend of changes in the values of samples after the jump starting from sample 50. It seems that during the operation of the device between samples 49 and 50, the moisture and temperature sensor probes were deactivated for some reason and properly activated afterwards. The first probe was analog, so it did not return any value, while the second one was digital and probably returned some incorrect (accidental) or previously recorded value in its reading register before being properly reset.

#### 2.2.4. Temporary Deviations

**Figure 6:**
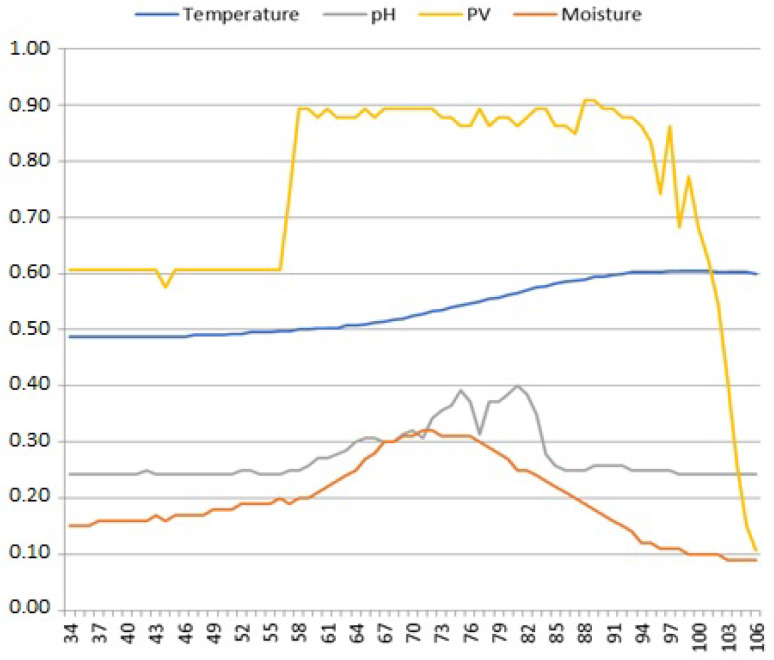
Bumps in the moisture and pH signals. Note the correlation of both signals, where the moisture signal reached its local maximum at sample 70 prior to the pH signal reaching its local maximum twice (samples 75 and 82); most likely, the end device was temporarily flooded.

Change points associated with temporary non-seasonal departures from the general daily signal trend occasionally took the form of local maxima in the analyzed signal plots, as shown in Figure 6. We refer to them further on as *bumps*. Taking into account the physical properties of soil measurement signals, the reason for such a signal pattern has its source in the course of the monitored phenomenon itself, external to the measurement system, rather than any momentary disturbances in its operation. The question remains as to whether bumps in soil measurement signals are really anomalies. However, their location and dimensions in the signal waveform seem reasonable, allowing for the possibility of eliminating them during time series cleaning as redundant data, from the point of view of their further use in data fusion in the cloud.

#### 2.2.5. Irregular Fluctuations

**Figure 7:**
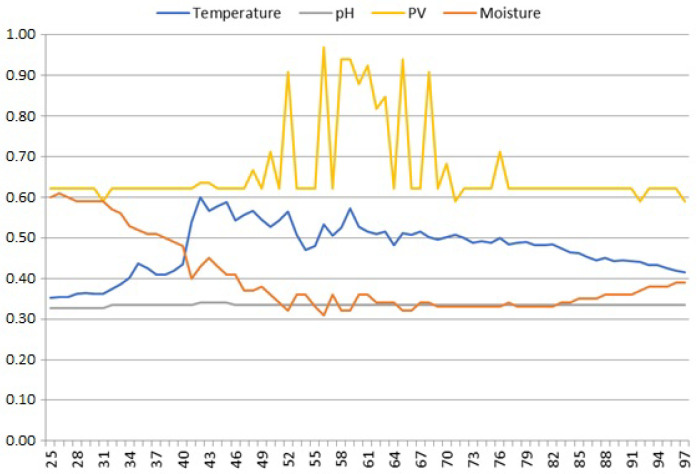
Instabilities in the temperature and moisture signals. Most likely, the temperature and moisture sensing probes were subject to small disturbances in the available power due to small variations in loads on the PV circuit caused by an undercharged battery.

Although the time series of our measured soil parameters should be stable over their daily periods, we encountered sporadic short-term fluctuations around their individual trend lines. One example would be the measurements shown in Figure 7, where oscillations in the PV signal (samples 40–76) are clearly correlated with the fading oscillations in M and T signals (samples 40–71). Such oscillations occurring in several places in the analyzed time series, often correlated internally with the PV signal, are further on referred to as *instabilities*.

## 3. Time Series Data Cleaning

The comprehensive interpretation of soil signals introduced in the previous section allowed us to assign meaning to particular patterns, which can be observed and labeled in the time series of our measurement data. With that, we could build a generic model, which may represent power gaps, absolute errors, peaks, jumps, bumps, and instabilities just by attributing to them specific parameters.

### 3.1. Anomaly Model

**Figure 8:**
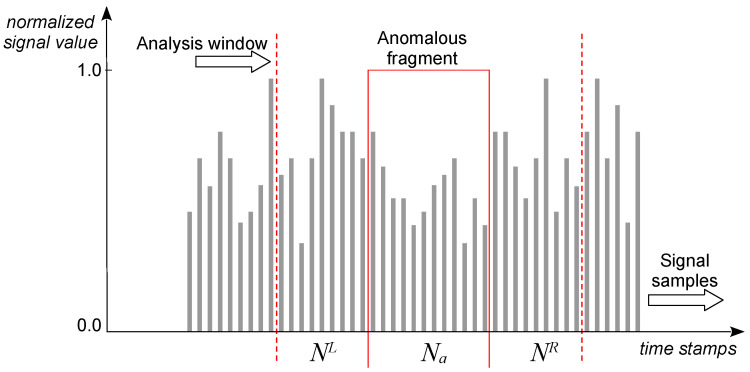
Generic anomaly model.

The model is quite straightforward to implement and is based on analyzing the relative metrics of adjacent fragments of data samples within a $W=N^{L}\cup N_{a}\cup N^{R}$ analysis window, as shown in Figure 8, where symbol a∈{(A)bsolute,(P)eak,(J)ump,(B)ump,(I)stabilities} represents a type of the anomalous fragment.

During the analysis, window *W* is moving along the entire 24 h portion of $N^{day}$ samples of each respective signal listed in Table 1, i.e., between two consecutive sunrises indicated by the corresponding PV voltage rises. Based on a good understanding of the physical nature of the anomaly under study, selecting appropriate sizes of the analyzed fragments $N^{L}$, $N_{a}$, and $N^{R}$, as well as defining related metrics for comparing the values of samples from these fragments, is not particularly difficult for the anomaly detector to compute. Moreover, we will argue later in Section 4 that heuristically set values of these parameters can be further optimized using various AI techniques.

### 3.2. Anomaly Detection

Several parameters and their values are selected for each specific anomaly, including the average $\mu^{N}$, maximum max(N), and minimum min(N) values of samples within a signal fragment *N* of interest, values $x_{a}^{fs}$ and $x_{a}^{ls}$ of the respective first and last samples of an anomalous fragment $N_{a}$, the total number $n_{a}^{LR}=\left|W-N_{a}\right|$ of samples complementing the anomalous fragment $N_{a}$ in window *W*, and various threshold values $th_{m}^{n}$ for comparing related metrics calculated for selected fragments of window *W*.

Below, we present how to adjust the model from Figure 8 to individual types of anomalies described in Section 2.2. The adjustment involves selecting specific parameters of window *W* and assigning them appropriate values. These values can be defined heuristically, according to the best knowledge of the signals’ human interpreter (sensor software developer), as well as optimized using AI methods. We will present a summary of both heuristic and optimized values further in Section 5, where we assess the quality of our anomaly detector based on the aforementioned model.

#### 3.2.1. Power Gaps

Upon determining the average daily number of samples $|N_{avg}^{day}|$ and taking into account possible deviations in $\Delta N^{day}$ from that average, due to fluctuations in the sampling rate caused by the sensor’s timer drift, the criterion for detecting power gaps may be stated formally as follows:

(1) $$\left|\frac{|N^{day}\left|-\right|N_{avg}^{day}|}{|N_{avg}^{day}|}\right|<th_{\Delta N^{day}},$$

where $th_{\Delta N^{day}}$ denotes a threshold for distinguishing misplaced samples from correct ones. In our rural IoT implementation, with a 10 min sampling period and a sporadic 6–15 min timer drift, we had $|N_{avg}^{day}|\approx 133\pm 25$ samples and $th_{\Delta N^{day}}=19\%$.

#### 3.2.2. Absolute Errors

Detection of all abnormal samples of this type in the analyzed series is straightforward and requires comparing the values of each individual sample, i.e., $|N_{A}|\;=\;1$, with the respective limits specified in Table 1.

#### 3.2.3. Peaks

An anomalous fragment $N_{P}$ representing a peak in T, M, or pH signal can be detected by comparing the values of samples in the left $N^{L}$ and right $N^{P}$ fragments of the $N_{P}$ fragment to the peak value $max(N_{P})$. Formally, this can be expressed as follows:

(2) $$\frac{\max(N_{P})}{\max(N^{L})}>th_{P},\;\frac{\max(N_{P})}{\max(N^{R})}>th_{P},$$

where $\max(N_{P})$ is the highest value in the $N_{P}$ fragment, $\max(N^{L})$ and $\max(N^{R})$ are the highest values in $N^{L}$ and $N^{R}$ fragments, and $th_{P}$ is a threshold value. An additional condition for peak detection is to check whether the relative difference in height between the left and right sides is not too great, i.e.,

(3) $$\left|1-\frac{\max\left(N_{P}\right)-x_{P}^{fs}}{\max\left(N_{P}\right)-x_{P}^{ls}}\right|<th_{P}^{LR},$$

where $x_{P}^{fs}$ and $x_{P}^{ls}$ are values of the first and last samples in the anomalous $N_{P}$ fragment, respectively, and $th_{P}^{LR}$ is a threshold value.

#### 3.2.4. Jumps

Jumps in T, M, or pH signal can be detected by comparing the values $x_{J}^{fs}$ and $x_{J}^{ls}$ of the boundary samples of the $N_{J}$ anomalous fragment and checking whether the signal is relatively flat around it. The following formula is used to compare the boundary values:

(4) $$g\left(x_{J}^{fs},x_{J}^{ls}\right)>th_{J}^{LR},$$

where *g* is a function defined as g(u,v)=(1+max(u,v))/(1+min(u,v)), and $th_{J}^{LR}$ is a threshold value. The flatness of fragments of length $n_{J}^{LR}=\left|N^{L}\cup N^{R}\right|$ around the anomaly is determined by the following formulas:

(5) $$\frac{\sigma_{L}}{|x_{J}^{fs}-x_{J}^{ls}|}<th_{J}^{dev},\;\frac{\sigma_{R}}{|x_{J}^{fs}-x_{J}^{ls}|}<th_{J}^{dev},$$

where $\sigma_{L}$ and $\sigma_{R}$ are the average deviations in the signal values from $x_{J}^{fs}$ and $x_{J}^{ls}$ calculated for the $N^{L}$ and $N^{R}$ fragments, respectively, and $th_{J}^{dev}$ is a threshold value.

#### 3.2.5. Bumps

An anomalous fragment $N_{B}$ representing a bump in T, M, or pH signal can be detected based on the following conditions, checking if they are met:

- A sufficiently small difference between the values of the first and last samples of the fragment containing the bump:
  (6) $$g\left(x_{B}^{fs},x_{B}^{ls}\right)<th_{B}^{LR},$$
  where $x_{B}^{fs}$ and $x_{B}^{ls}$ are the signal values at the boundaries of the $N_{B}$ anomalous fragment, *g* is a function defined above, and $th_{B}^{LR}$ is a threshold value;
- A sufficiently small difference between the value of the first sample in $N_{B}$ and the signal mean value in the fragment on the left side of the bump:
  (7) $$g\left(x_{B}^{fs},\mu^{L}\right)<th_{B}^{bound},$$
  where $\mu^{L}$ is the mean signal value in the $N^{L}$ fragment, and $th_{B}^{bound}$ is a threshold value;
- A sufficiently small difference between the right boundary value of the bump and the signal mean value in the fragment on the right side of the bump:
  (8) $$g\left(x_{B}^{ls},\mu^{R}\right)<th_{B}^{bound},$$
  where $\mu^{R}$ is the mean signal value in the $N^{R}$ fragment;
- A sufficiently large difference between the mean signal mean in the fragment on the left side of the bump and the signal mean value in the fragment containing the bump:
  (9) $$g\left(\mu^{B},\mu^{L}\right)>th_{B},$$

where $\mu^{B}$ is the signal mean value in the $N_{B}$ fragment, and $th_{B}$ is a threshold value.

#### 3.2.6. Instabilities

Instability in T, M, or pH signal is detected in fragments where there are many sharp maxima or minima, i.e., when the normalized sum of the curvature of the extrema expressed by the second derivative of the signal exceeds a threshold value $th_{I}$ in the $N_{J}$ anomalous fragment:

(10) $$\frac{\sum_{i=k+1}^{k+|N_{I}|}\max\left(-x_{i}^{\prime }x_{i+1}^{\prime },0\right)x_{i}^{\prime \prime }}{\mu }>th_{I},$$

where $x_{i}^{\prime }=x_{i+1}-x_{i}$ is the first differential of the signal at point *i*, $x_{i}^{\prime \prime }=x_{i+1}^{\prime }-x_{i}^{\prime }$ is the second differential of the signal at point *i*, and μ is the mean value of the samples collected throughout the entire growing season.

### 3.3. Cleaning Operators

**Figure 9:**
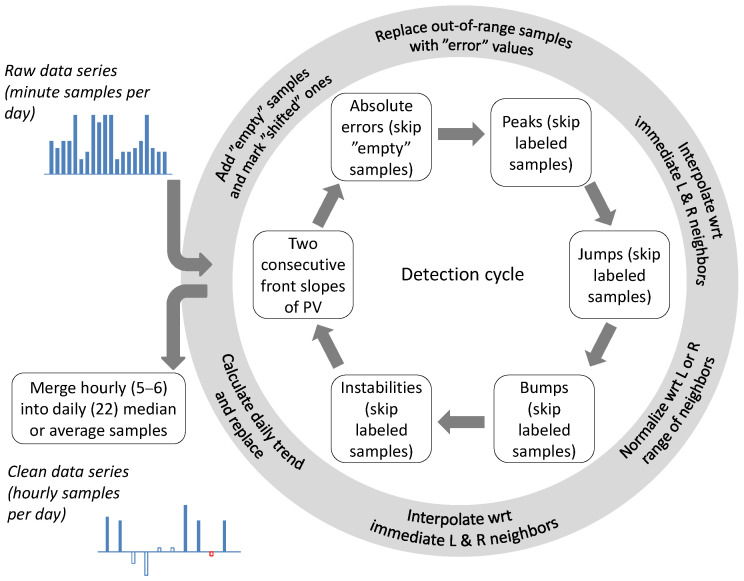
Daily time series data anomaly detection and cleaning. After cleaning, minute samples are aggregated into hourly samples.

As argued above, our constrained end devices are limited in two ways. Firstly, they have a maximum available daily supply of electrical energy needed for reading data from sensor probes and recomputing them for transmission. Secondly, they have to minimize the number of frames to be sent. For that reason, they should implement the “best effort” approach so as not to “overclean” the portion of data to be uploaded to the UAV: recomputing the data to make them fit in the available Δt fly-over window, and at the same time retaining in the transmitted data frames all essential information that may be needed for fusion in the cloud instance when combined with data from other sensors. Therefore, each sensor must decide for itself whether any suspicious-looking signal fragments should be repaired or simply marked as such before sending. Local data repairs may be carried out only when the samples being removed or corrected are indeed erratic and may disturb fusion with data from other sensors after delivery to the cloud. However, due to limited power supply, not much calculation can be performed in this regard by the sensor. In other words, daily portions of samples should be aggregated to eliminate redundant data, but incorrect values should be fixed or marked if possible, based on the analysis of their local context. To this end, we adopted a heuristic approach, as outlined in Figure 9. The purpose of the operation presented there is to remove or correct 10 min samples of signals identified as erroneous, before aggregating them into hourly samples.

The rationale for the above scheme is as follows:

1. Samples misplaced by power outages may have correct values, so they have to be marked as “shifted”. It may be implemented, for example, by inverting the sign bit of each marked sample value. Finding them requires calculating Formula (1) for each two consecutive front slopes of the PV signal, and if needed, a missing number of “empty” samples for each signal, T, M, and pH, is added. Although the end device could try to determine the locations of the missing samples by examining disturbances in the trends of other predictable signals, e.g., signal T, due to its stable daily periodicity discussed in Section 2, for some implementations of the end device, it may still be too power-costly to implement. In our current implementation of rural IoT, we skipped that and found that the fusion of series from multiple sensors performed in the cloud gave better results; “empty” and “shifted” values of minute samples may be considered as “misleading” data when merging them into hourly (median or average) samples by the sensor for further resolution on the cloud, where they can certainly be handled more accurately than on a local end device, without adding any extra bandwidth load.
2. After detecting power gaps and marking samples of each of the four signals as “shifted” or “empty”, the end device continues detecting anomalies only in signals T, M, and pH. This is because variability in the PV signal, as argued in Section 2, is caused by charging of the device’s battery; in fact, it shows no anomalies worth analyzing and correcting, except for proper handling of power gaps that affect the other three. So the next step in Figure 9 is the detection of “absolute errors”, i.e., minute samples whose values are outside the allowed ranges specified in Table 1. Out-of-range values of minute samples may not be taken into account when merging minute samples into the hourly ones; therefore, they are labeled as “error” samples. Similarly, previously inserted “empty” samples will also be disregarded in the merging process. Note that marking “error” samples does not affect “shifted” samples with correct values. During fusion later in the cloud, the “shifted and error” samples may eventually be properly time-stamped and set a correct value.
3. The next step should be the detection of abrupt changes, i.e., “peaks” and “jumps”. This order comes from the fact that according to their physics analyzed in Section 2, all changes in our soil signals should be smooth and gentle. Detection of abrupt changes indicates the occurrence of anomalies in the measurement process itself; thus, signal values in any fragment identified as anomalous are in error. The respective samples are replaced by samples with interpolated values of their neighbors not marked as “empty”, “shifted”, or “error”.
4. After “peaks” and “jumps”, less abrupt signal changes such as “bumps” are handled. As discussed earlier, these anomalies are related to the occurrence of a local maximum in a relatively larger portion of samples and detected. If needed, the “bump” fragment of samples is slightly more flattened by calculating its new values based on the average values of samples from both its left and right sides. As before, neighbor samples marked as “empty”, “shifted”, or “error” are not taken into account.
5. Finally, “instabilities” are detected and samples from their anomalous fragments are replaced with the signal trend samples calculated as a daily moving average.

Merging minute samples into hourly ones is straightforward. For each cleaned signal in the daily multivariate series of $N_{avg}^{day}=133$ samples, consecutive portions of

(11) $$N_{hr}=\left|N_{avg}^{day}\right|÷\left\lfloor \frac{\tau \cdot |N_{avg}^{day}|}{60}\right\rfloor$$

minute samples, where τ=10 is a minute sampling period, are aggregated into one hourly sample as follows:

1. The $N_{hr}$ portion may contain correct (unlabeled) and “error” samples. If at least half of them are correct, the aggregated hourly sample is calculated as their average or median; otherwise, it is labeled as “error”. Note that any other combination of unlabeled and labeled samples in $N_{hr}$ is not possible.
2. $N_{hr}$ may contain “shifted” samples, of which some may be marked additionally as “error”. If at least half of the “shifted” but correct samples are present, the aggregated hourly sample is calculated as their average or median; otherwise, it is labeled as “shifted and error”. Note that the absolute values of “shifted” samples are considered correct and are needed later on for data fusion in the cloud.
3. If $N_{hr}$ contains at least half of samples marked as “empty”, the aggregated hourly sample is also marked as “empty”; otherwise, the aggregated hourly sample is either calculated as the average or median of the complement samples or marked as “error”—depending on whether the rest of the portion is marked only as “shifted” or “shifted and error”.

The above-described conservative approach to the aggregation of minute data was dictated, on the one hand, by the desire to limit the computational effort of the sensor, and on the other hand, by the assumption that even in the case of aggregation burdened with an error, the fusion of data from multiple sensors will be able to correct it.

## 4. Improvement of the Anomaly Model

A comprehensive analysis of the physics of rural IoT soil signals helped us to identify and understand several key types of anomalies that may be observed in time series of measurement data. Based on that understanding, we were able to define a general model of these anomalies and determine parameters that govern their informed and unbiased detection for cleaning. However, the heuristic values for these parameters based on the understanding of the anomalies specified in the previous section may not necessarily result in series being optimally cleaned before being sent on-board the UAV. The question arises as to whether all threshold and range values used to calculate formulas specified in Section 3.2 could yield better results in terms of the quality of the cleaned time series. Solving this task using the relatively small set of real measurement data that we had at our disposal was rather difficult compared to methods that, although capable of handling small amounts of labeled data, still require larger sets of unlabeled data [21].

### 4.1. Synthetic Data Generation

A data-free approach described in [22] allowed us to turn the low volume of measurement data to our advantage. This operation, however, was possible only after understanding the physics of anomalies. With that in mind, we first constructed reference (ideal) signals based on the originally recorded time series data. All anomalies visible in individual daily intervals determined by subsequent sunrises were corrected: abrupt and gentle signal deformations were smoothed out automatically, whereas each missing or misaligned daily portion of samples was manually replaced with a complete sequence of samples with no visible power gaps by copying them from the nearest preceding or following day. The rationale for this operation was the very moderate dynamics of all three signals T, M, and pH, as specified in Table 1. Next, we generated hundreds of mutant time series by injecting into the reference (ideal) series various anomalies of all six types defined formally in Section 3.2, with values of individual parameters changing randomly. Anomalies were injected realistically; i.e., for each anomaly and signal type, a randomly selected week was subject to local mutations within one of its days ($N^{day}$ fragments). Random gaps were added to the signals, with up to 5% of missing samples on average to imitate power gaps.

This process is described formally by Algorithm 1. The random selection of anomaly positions took into account the following principles, to keep the anomalies fairly realistic:

- For a given device, gaps are inserted at the same positions for all signals T, M, pH, and PV;
- The positions of anomalies other than gaps are not synchronized among signals;
- A maximum of one jump per day is inserted; its edge is selected at random;
- A jump edge is placed randomly within any $N^{day}$ fragment and the durable change point samples are continued until the sunset sample or inserted before the edge starting from the sunrise sample;
- Bumps and jumps do not overlap;
- There is no significant difference between the average value of samples before and after a bump; i.e., bumps are not injected on the steep slopes of time series;
- Some minimum distance between an instability and a jump or bump is preserved;
- Peaks do not overlap with other anomalies;
- There is no significant difference between the average value of samples before and after a peak; i.e., peaks are not injected on the steep slopes of time series;
- Peaks and instabilities are not adjacent to gaps; i.e., there are some samples before and after a peak or instability.

**Algorithm 1** Injecting anomalies to a reference time series.1:**function** InjectAnomalies(ref,num,g,j,b,i,p)2:    ▹ **Input parameters:**3:        ▹ref—reference time series4:        ▹num—number of series generated on the basis of ref5:        ▹*g*—percentage o missing samples in the output series6:        ▹j,b,i,p—number of jumps, bumps, instabilities, and peaks7:    ▹ **Output:**8:    ▹*S*—a set of generated series with anomalies9:    S=∅10:    **for** n=1,…,num **do**11:        s←Gaps(ref, *g*)12:        s←Jumps(*s*, *j*)13:        s←Bumps(*s*, *b*)14:        s←Instabilities(*s*, *i*)15:        s←Peaks(*s*, *p*)16:        S←S∪{s}17:    **end for**18:    **return** *S*19:**end function**

The shapes and sizes of the inserted anomalies depend on their parameters. The values of these parameters are randomly selected, taking into account predefined limitations on their range of variability. Bumps are approximated by a sine wave. Table 2 presents parameters, which are randomly chosen for each anomaly instance.

The measurement data generated in this way, although synthetic, contained anomalies realistically related to the physical properties of the measurement processes.

### 4.2. Parameter Optimization

**Figure 10:**
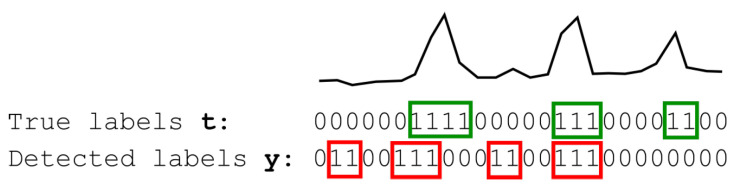
Exemplary labeling of anomalies as “true” or “detected”. Sequence *t* of ground truth labels shows anomalies in a given (analyzed) signal marked in green, whereas sequence *y* of labels is generated by the anomaly detector (in red). Anomalous samples are indicated by 1 s; otherwise, they are correct and indicated by 0 s. In this example, the first anomaly marked in green was partially recognized because its red counterpart only partially matches it, while the second anomaly marked in green perfectly matches its red counterpart. Moreover, the third anomaly marked in green was not detected at all, and the other two anomalies marked in red were falsely detected.

Training and testing of the anomaly detector were performed using mutant multiseries, generated by injecting into individual reference signals all the anomalies specified in Section 2 with randomly selected parameters. Samples in each time series anomaly fragment $N_{A}$ were labeled with 1 s, whereas all others were labeled with 0 s; we distinguish *true* and *detected* anomalies as outlined in Figure 10.

At this stage of the experiment, our task was to optimize the parameter values of individual anomalies so that the detected anomalies were best matched to the true ones. Two optimization criteria were used. One is the *sample error*:

(12) $$E_{smp}=\frac{\left|t⊕y\right|}{\left(|t|+\min(|y|,|t|)\right)}$$

calculated as the normalized sum of samples with different “true” and “detected” anomaly labels. t⊕y is an exlusive-or operation between samples, and |.| is the sum of the elements of the sequence. The other is the *sequence error*:

(13) $$E_{sqn}=\frac{\sum_{j=1}^{M}E_{y}\left(p_{j}\left(t\right)\right)+\sum_{j=1}^{K}E_{t}\left(p_{j}\left(y\right)\right)}{M+\min(K,M)},$$

where *M* and *K* denote the respective numbers of “true” and “detected” anomalies, $E_{y}\left(p_{j}\left(t\right)\right)$ is the error coverage metric of the *j*th true anomaly matching $p_{j}\left(t\right)$ labels of detected anomalies *y*, and $E_{t}\left(p_{j}\left(y\right)\right)$ is the coverage metric of the *j*th detected anomaly matching $p_{j}\left(y\right)$ labels of true anomalies *t*. The coverage error is calculated as follows:

(14) $$E_{q}\left(p\right)={\left(1-|p\cap q|/|p|\right)}^{2},$$

where |p∩q| is the number of samples labeled with *p* and *q*, respectively. The min() function was used in error Formulas (12) and (13) to counteract the trend of excessive increases in the number and scope of false anomaly detections, if $\sum_{i}y_{i}$ and *K*, respectively, were used instead. For the situation shown in Figure 10, it may be readily seen by calculating Formulas (12) and (13) that $E_{smp}=0.50$ and $E_{sqn}=0.56$. Due to the fact that during the experiment, both criteria gave different results for various anomalies and sensors, the average error $E=(E_{smp}+E_{sqn})/2$ was assumed as the loss function for the optimization algorithm.

Our anomaly detector was trained with datasets of three randomly selected sensor locations; for each sensor, two time series of each mutated signal T, M, and pH were used, respectively. The training process involved tuning individual detection parameters for each of the four anomalies described in Section 3.2, starting from some initially set values. Consequently, a total of 12 independent optimization processes for all four anomalies and three signals T, M, and pH were performed in parallel using the simulated annealing (SA) method. The reason for adopting this method was a relatively small number of training examples requiring the use of heuristic knowledge along with a small number of parameters requiring tuning based on the data, typically from two to five, depending on the anomaly type. In total, for all our anomalies, the parameter vector (Θ) contained 15 parameters.

The main idea of the SA method is to allow the solution to deteriorate temporarily in order to avoid stagnation at a local minimum. For this purpose, the T meta-parameter is used, which determines the amount of exploration. In the initial optimization period, exploration should be high and then decrease due to the improvement of the solution and the increasing probability of locating the current solution near the global minimum. In our experiment, the T meta-parameter was used to determine both the probability of accepting a new solution and the scope of choosing a new solution.

The probability of accepting a new solution was determined using a standard formula:

(15) $$P_{acc}=1/(1+\exp\left(\Delta E/cT\right),$$

where *c* is a coefficient discriminating influence of the T parameter on the probability of acceptance and the scope of selection of a new solution, and  $\Delta E=E_{curr}-E_{prev}$ is the difference between the current and previous solution values. If *E* is a loss function, i.e., the minimum of the criterion function is sought, ΔE<0 means solution improvement. During optimization, the value of T is reduced by multiplying it by some change rate value $w_{T}<1$, thus reducing the probability of accepting worse solutions in favor of solutions that are better than the previous ones. In the extreme case, when T is close to zero, only solutions better than the previous ones are accepted. In our experiment, we made the change rate dependent on the planned number of optimization epochs so that T reached value $T_{min}$ in the last epoch, by calculating

(16) $$w_{T}=\exp\frac{\log(T_{min}/T)}{n_{e}+1},$$

where $n_{e}$ is the number of epochs. The scope for selecting a new solution was also systematically reduced during optimization in proportion to T. A new solution was generated randomly in the vicinity of the current solution by adding a random vector with a length proportional to T and normalized with respect to the initial parameter values. In that way, different scales of individual parameters were taken into account, i.e., $\Theta^{\prime }=\Theta +\Delta$, where $\Delta =N\left(\mu =0,\sigma^{2}=T\right)⊙\Theta_{h}$, N is a 15-dimensional normal distribution, $\Theta_{h}$ is a vector of the heuristically determined values of anomaly parameters, and ⊙ denotes an element-by-element multiplication operator.

## 5. Experimental Results

**Figure 11:**
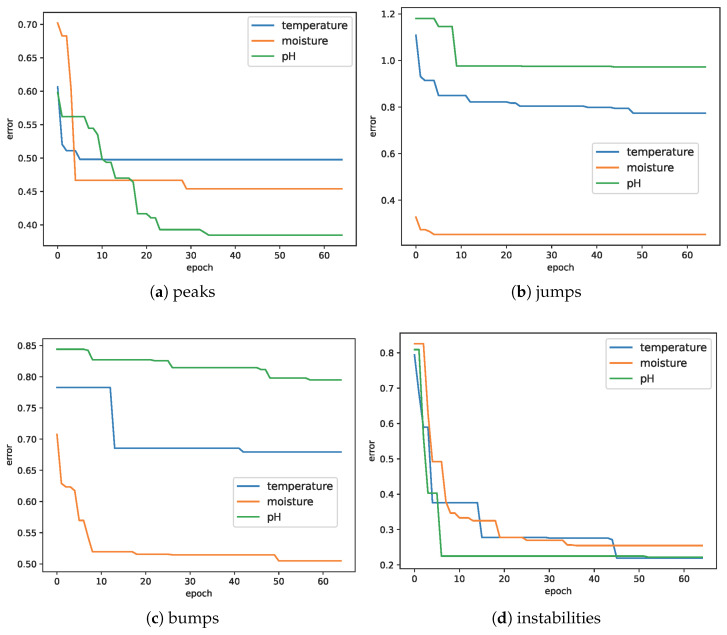
Reduction in average error $E=(E_{smp}+E_{sqn})/2$ calculated on the basis of training data during parameter optimization. Taking into account both $E_{smp}$ and $E_{sqn}$ helps to avoid local minima during optimization.

Plots of the average error *E* changes during the optimization of detection parameters of all four anomaly types are presented in Figure 11.

The detailed test error results for the testing datasets we obtained with the optimized values of anomaly parameters vs. the results obtained with their initially set (heuristic) values are presented in Table 3. For higher credibility, testing was performed with datasets for the other four sensors; it may be seen that results after optimization are clearly better compared to results for the initial parameter values.

The heuristic and optimized values parameter values of each type of anomaly for individual T, M, and pH signals are presented in Table 4. The heuristic values in the upper row of Table 4 were common to all signals (T, M, and pH), whereas the three lower rows contain the final values for each physical parameter after the optimization process.

With the sets of initial and optimal values of the anomaly parameters, the quality of our anomaly detector could finally be evaluated. For each reference signal, ten anomalous series were chosen, different from those used for parameter optimization. These series were first cleaned using the initial (heuristic) values specified in Section 3.2. Then, the same anomalous series were cleaned using the optimized values of our anomaly model parameters. For evaluating their quality, we used a quality metric based on the concept of measuring the distance between two series.

### 5.1. Time Series Distance Metrics

To determine the dissimilarity between two time series, various distance measures may be applied [23]. The choice depends on the properties of data and the aim of a given task [24]. After analyzing the possibilities of individual metrics, we chose a feature-based distance measure. In consequence, we represented time series as feature vectors, for which we could calculate a distance between them. The rationale for this approach was twofold—it would be possible to compare series of different lengths, and with properly selected features, the differences between ideal and anomalous series could be highlighted. Defining a feature-based distance measure capable of distinguishing ideal reference time series in a feature space from the ones with anomalies required us to define a set of features sensitive to the anomalies present in our data. Two sets of features were considered in that regard. One contained features describing long series, i.e., sequences of data representing at least several days. The other included parameters describing daily series. For each set of daily features, its minimum (min), maximum (max), and mean (*m*) over the whole sequence were calculated and added to the first set.

The sensitivity of the parameters proposed initially was treated as a hypothesis, which needed verification on the basis of the generated (mutant) data. Our aim was to identify features showing significantly different distributions between ideal reference sequences and their counterparts with injected anomalies. Various statistical tests may be applied for this evaluation, depending on the type of features and the target, for example, the non-parametric Kolmogorov–Smirnov test. Testing the set of features was a multiple testing problem, which carried the risk of falsely recognizing some features as relevant. To prevent that, we applied the Benjamini–Hochberg procedure to control the false discovery rate (FDR) [25].

### 5.2. Quality Assessment of Cleaned Data

All series were split to one-week segments. In this way, a set of 96 one-week reference series and 96 volumes of one-week series with anomalies were created. All series were then represented by feature vectors composed of 237 parameters, 79 for each T, M, and pH signal, respectively. These parameters were picked heuristically after visual analysis of the available data, as discussed before. The usefulness of all 237 features was evaluated by applying the aforementioned Kolmogorov–Smirnov test with the Benjamini–Hochberg correction for an FDR level of 0.01 [26]. The process of creating a set of anomalous series and selecting features on the basis of this data was repeated 100 times. In each iteration, the best features were identified and selected for inclusion in the final feature set. The last step of feature selection was removing the highly correlated ones. The final set of 70 parameters included *multi-day* and *one-day features*. The latter were calculated for each day in a one-week window and then its weekly minimum (min), maximum (max), and average (*m*) parameters were incorporated into the feature vector. Table 5 presents the set of selected features. The type of aggregation of the selected feature is specified in the table within square brackets “[]”.

The same procedure was performed for series with anomalies of one type. In this way, parameters particularly relevant to the selected types of anomalies were also identified; e.g., the top three features for “peaks” were as follows: the total number of changes in slope direction (signal M), absolute differences between subsequent values (signals T, M, and pH), and standard deviation *s* of local maxima (signals T and pH). The implementation of feature extraction and selection utilized the tsfresh Python package (ver. 0.20.1) [27].

**Figure 12:**
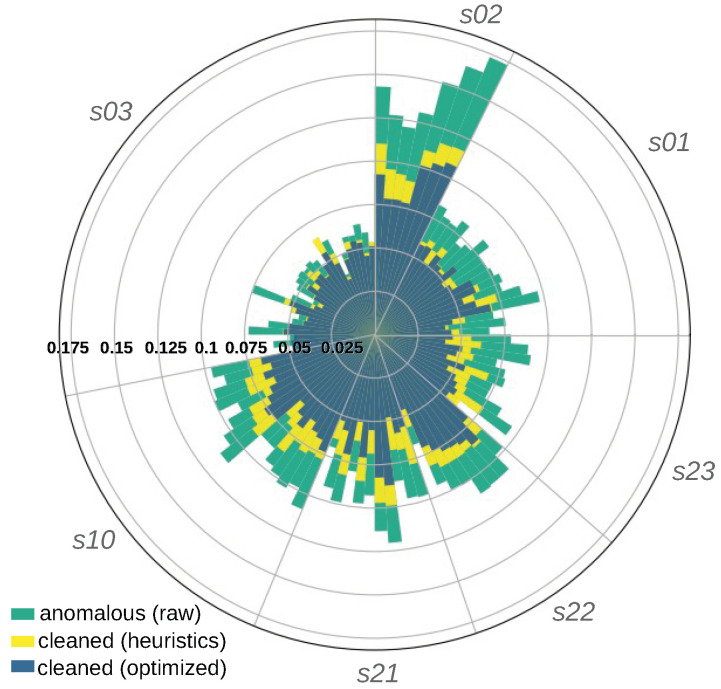
Distances from the reference series. With heuristic values of the anomaly parameters, the distance to the reference series was reduced by 16.34% on average, whereas after their optimization, it decreased by 24.95% on average.

After cleaning the series with two sets of values of parameters of the four considered anomalies (“peaks”, “jumps”, “bumps”, and “instabilities”), the distances between anomalous and cleaned series were calculated and compared with the distances between the anomalous and the reference series. The length of the reference series varied from 7 to 27 weeks depending on device. While calculating the distances, all signals were split into one-week subseries. The rationale for that was that the UAV collects data every few days, so cleaning had to be performed accordingly for several-day time series. The total number of one-week reference samples was 96. Each of them was compared with 10 anomalous subseries. Each of the 960 anomalous subseries was compared to its two counterparts cleaned with both heuristic and optimized values of anomaly parameters. The results clearly show improvement in the cleaning procedure with the latter. Figure 12 illustrates that for all seven (*s01–s03*, *s10*, *s21–s23*) devices, the distances were averaged across 10 mutants in every week of the entire lifetime of each sensor.

## 6. Conclusions

The novelty of our research lies in adopting a comprehensive approach to developing an effective method that can overcome several inherent limitations of nomadic computing with a UAV. They are related both to using constrained end devices for data collection and cleaning, and to the difficulty of acquiring, in an economically viable time, a sufficiently large volume of real physical data for training ML models for performing the task of classifying anomalies in time series of measurement data when implemented on these devices.

In our method, two complexity levels of using AI can be distinguished. The lower one concerns the implementation of an anomaly detector that should consume as little energy reserve of the sensor as possible. For this purpose, we used simple heuristic processing, which, after additional optimization of its code, could be limited to several hundred arithmetic operations per analysis window. Alternatively, our detector could be programmed as a simple feedforward neural network. The latter, however, would require the use of at least several layers of neurons and about several thousand arithmetic operations per window. Moreover, its code cannot be further optimized to relieve the end device of the computational load it brings. The higher complexity level of using AI in our scheme concerns the optimization of the anomaly detector parameters, which, due to the relatively low volume of data, must be performed in a cloud instance supporting the rural IoT ecosystem. This, in turn, requires the generation of synthetic data to augment the data necessary to train an optimized version of the detector, or the fusion of data from multiple sensors. In this paper, in fact, we combined both: we created reference (ideal) signals based on time series from all sensors, and then generated synthetic data by introducing random anomalies into each reference signal, the physical features of which were extracted using our comprehensive approach from all data acquired over the entire growing season. Due to the practically unlimited computing resources of the supporting cloud instance, various popular AI techniques can be used to generate (train) our optimized anomaly detector. In the experiment described in Section 5, we used a simple simulated annealing technique.

By taking advantage of the fact that the physics of T, M, and pH signals read from the soil by sensor probes is relatively simple to capture and explain, we were able to identify quite a small set of classes of anomalies occurring in these signals. Each class can be explained and characterized with a well-defined vector of parameters. Heuristic selection of their values, made a priori by a knowledgeable sensor developer, enabled the direct implementation of a resource-efficient anomaly detector on a constrained device. We also demonstrated that these values can be effectively optimized using straightforward techniques to obtain even better detection results. It is worth noting that the mechanism we used for generating mutant time series with anomalies with randomly changing parameters was, in fact, an implementation of a simple digital twin of the measurement processes of soil parameters. All activities described in this paper, from collecting and storing data from soil sensors to visualization and interpretation, up to the optimization of the anomaly detection and localization models, were supported by a tech stack developed by us on the TASKcloud computing cloud operated by Gdańsk Tech [28].

## Figures and Tables

**Table 1:** Physical properties of the time series data of the four measured soil parameters. The allowable range of variation for each parameter was known in advance so that the sensor software could identify erratic samples and attempt to correct or eliminate them before transmitting data to the UAV. All time series plots containing anomalies analyzed in this paper have values of samples normalized to *T*_*max*_ = 40 °C, M_*max*_ = 100%, pH_*max*_ = 14.0, PV_*max*_ = 6.6 V.

Signal	Unit	Range	Physical Quantity Measured	Change	Seasonality
Temperature (T)	°C	[0, 40]	Resistance of a thermistor placed in the ground (approx. 0.5 m)	mild trend	daily
Moisture (M)	%	[10, 80]	Capacity of the capacitor in the form of a printed circuit board placed in the ground (approx. 0.2 m)	slow trend	non-daily
Acidity–alkalinity (pH)	—	[3.0, 9.0]	Electromotive force of a cell composed of a glass indicator electrode and a reference electrode placed in the ground	almost constant	no periodic fluctuations
Solar irradiation (PV)	V	[0.0, 6.6]	Open-circuit voltage of the PV cell	rapid	drop (rise) at sunset (sunrise)

**Table 2:** Anomalies’ shape parameters.

Anomaly	Parameter	Description
Power gap	G_width	gap width
Jump	J_slope_width	slope width
	J_direction	whether the values jump up or down
	J_height	jump height
	J_right	whether the modified segment is before or after the jump
Bump	B_width	bump width
	B_height	bump height
	B_noise	noise vector of length B_width
Instability	I_width	instability width
	I_delta	noise vector of length I_width
Peak	P_width	peak width
	P_max	maximum value of the peak
	P_loc	location of the peak maximum
	P_vals	remaining (other than the maximum) values of the peak

**Table 3:** Average error *E* for the initial and optimal parameters (testing datasets); it may even be greater than 1.0 in the case of a large number of false positive detections.

Parameters	Error	Peaks			Bumps			Jumps			Instabilities		
		T	M	pH	T	M	pH	T	M	pH	T	M	pH
initial	$E_{smp}$	0.75	0.69	0.71	1.01	0.8	1.0	1.75	0.47	0.99	0.76	0.82	0.88
	$E_{sqn}$	0.69	0.6	0.61	1.03	0.79	0.99	2.52	1.46	0.97	0.82	0.86	0.91
	*E*	0.72	0.65	0.66	1.02	0.8	1.0	2.14	0.97	0.98	0.79	0.84	0.9
optimized	$E_{smp}$	0.62	0.56	0.47	0.96	0.91	1.0	0.92	0.39	1.0	0.26	0.38	0.28
	$E_{sqn}$	0.47	0.4	0.31	0.97	0.83	0.99	0.83	0.42	1.0	0.16	0.29	0.25
	*E*	0.55	0.48	0.39	0.97	0.87	1.0	0.88	0.41	1.0	0.21	0.34	0.27

**Table 4:** Parameter values of the anomaly model.

Parameters	Peaks				Bumps					Jumps				Instabilities	
	$|N_{P}|$	$n_{P}^{\mathrm{LR}}$	${\mathrm{th}}_{P}$	${\mathrm{th}}_{P}^{\mathrm{LR}}$	$|N_{B}|$	$n_{B}^{\mathrm{LR}}$	${\mathrm{th}}_{B}$	${\mathrm{th}}_{B}^{\mathrm{bound}}$	${\mathrm{th}}_{B}^{\mathrm{LR}}$	$|N_{J}|$	$n_{J}^{\mathrm{LR}}$	${\mathrm{th}}_{J}^{\mathrm{LR}}$	${\mathrm{th}}_{J}^{\mathrm{dev}}$	${\mathrm{th}}_{I}$	$|N_{I}|$
initial (T, M, pH)	30	15	0.75	0.90	75	75	0.54	0.21	0.15	105	15	0.60	0.45	0.03	22.5
optimized (T)	12	11	0.46	0.78	46	71	0.58	0.20	0.14	71	6	0.077	0.21	0.038	14
optimized (M)	16	12	0.21	1.26	37	115	0.93	0.18	0.25	81	15	0.65	0.12	0.051	11
optimized (pH)	19	27	0.18	1.21	97	76	0.50	0.22	0.15	90	14	0.71	0.36	0.034	12

**Table 5:** The set of selected features.

MULTI-DAY FEATURES	
**Feature**	**Signal**
series length divided by the maximum possible samples per segment	T
standard deviation (SD)	T, M, pH
maximum value	T, M, pH
kurtosis	T, M, pH
percentage of values greater than the mean value	T
percentage of values greater than SD from the mean value	T, M, pH
mean, SD, and max of the absolute differences between subsequent values	T, M, pH
variation coefficient	T, pH
relative number of changes in slope direction	M
mean of local maxima	M
SD of local maxima	T, M, pH
SD of local minima	T, pH
mean and SD of the distance between consecutive local maxima	T, M
mean of the distance between consecutive local minima	T, M
SD of the distance between consecutive local minima	M
mean of the distance between local minima and the nearest subsequent maxima	M
SD of the distance between local minima and the nearest subsequent maxima	T
mean and SD of the distance between local maxima and the nearest subsequent minima	T
**ONE-DAY FEATURES**	
**Feature**	**Signal [aggregation]**
SD	T, M, pH[*m*, max]
maximum value	T[*m*], M[*m*], pH[*m*]
relative position of the first maximum	pH[min], T[max]
relative position of the last maximum	M[*m*, max], pH[max]
relative position of the first minimum	T[max], M[*m*]
relative index of time series where 50% of the mass lies on the left	pH[min, max, *m*], T[min, *m*]
relative number of changes in slope direction	M[min, max]
variation coefficient	T, pH[max, *m*]

## Data Availability

The original data presented in the study are openly available in IEEE DataPort at https://doi.org/10.21227/0j1h-ew11.

## Abbreviations

The following abbreviations are used in this manuscript:

FDR	False Discovery Rate
LoRaWAN	Long-Range Wide-Area Network
LiPo	Lithium Polymer
M	Moisture
MCU	Microcontroller Unit
pH	Potential of Hydrogen
PV	Photovoltaic
RAT	Radio Access Technology
SA	Simulated Annealing
T	Temperature
UAV	Unmanned Aerial Vehicle
